# Efficacy of Water-Soluble Pearl Powder Components Extracted by a CO_2_ Supercritical Extraction System in Promoting Wound Healing

**DOI:** 10.3390/ma14164458

**Published:** 2021-08-09

**Authors:** Minting Liu, Junjun Tao, Hongchen Guo, Liang Tang, Guorui Zhang, Changming Tang, Hu Zhou, Yunlong Wu, Huajun Ruan, Xian Jun Loh

**Affiliations:** 1 Fujian Provincial Key Laboratory of Innovative Drug Target Research and State Key Laboratory of Cellular Stress Biology, School of Pharmaceutical Sciences, Xiamen University, Xiamen 361102, China; 32320201154093@stu.xmu.edu.cn (M.L.); huzhou@xmu.edu.cn (H.Z.); wuyl@xmu.edu.cn (Y.W.); 2 Zhejiang Fenix Health Science and Technology Co., Ltd., Zhuji 311800, China; junjunt@126.com (J.T.); jessenghc@163.com (H.G.); TangL_1234562021@163.com (L.T.); zgr77129219@163.com (G.Z.); tang1chang2ming@163.com (C.T.); 3 Institute of Materials Research and Engineering, Agency for Science, Technology and Research, 2 Fusionopolis Way, Singapore 138634, Singapore

**Keywords:** pearl powder extract, CO_2_ supercritical extraction system, wound healing

## Abstract

Pearl powder is a biologically active substance that is widely used in traditional medicine, skin repair and maintenance. The traditional industrial extraction processes of pearl powder are mainly based on water, acid or enzyme extraction methods, all of which have their own drawbacks. In this study, we propose a new extraction process for these active ingredients, specifically, water-soluble components of pearl powder extracted by a CO_2_ supercritical extraction system (SFE), followed by the extraction efficiency evaluation. A wound-healing activity was evaluated in vitro and in vivo. This demonstrated that the supercritical extraction technique showed high efficiency as measured by the total protein percentage. The extracts exhibited cell proliferation and migration-promoting activity, in addition to improving collagen formation and healing efficiency in vivo. In brief, this study proposes a novel extraction process for pearl powder, and the extracts were also explored for wound-healing bioactivity, demonstrating the potential in wound healing.

## 1. Introduction

Trauma is a very common disease, which can be divided into two categories: acute wounds (surgery, trauma, abrasions and burns) and chronic wounds (diabetic foot ulcers) [1]. Chronic wounds caused by chronic diseases such as diabetes or peripheral vascular disease can lead to abnormal wound healing. After acute injury (such as peeling or large-scale thermal injury from surgery), the function of the skin organ is lost, which makes organisms susceptible to infection, thermal disorders and fluid loss [2]. Even with a simple wound after a minor operation (such as an outpatient operation), patients may develop scars and therefore require proper attention and care [3]. Wound healing embodies a dynamic and instantaneous reaction of the body to tissue injury with the objective of repairing anatomical continuity, structure and function. The success or failure of this complex cascade of events is defined largely by the competence of the host’s immune system. Sepsis exemplifies one of the most challenging risks to effective wound healing. This is where antimicrobial agents are extremely crucial for wound healing [4,5,6,7]. The wound healing process is strictly regulated by many growth factors and cytokines released from the wound site. It can be classified into three consecutive and overlapping processes: in the early stage of injury, platelets play a hemostatic function, and inflammatory cells engulf necrotic tissue and release various factors.; next is the proliferation process that leads to tissue recovery: keratinocytes migrate and differentiate into the epidermis and fibrosis. Cells migrate and differentiate to synthesize collagen fibers and other proteins to form granulation tissue; the third step is tissue remodeling, whereby the fibrin clot formed in the early stage of inflammation gradually transforms into collagenous tissue that is rich in type III collagen and granulation tissue, and subsequently is replaced by collagen scars, which are mainly type I collagen [8].

In recent years, autologous platelet-rich plasma has been proposed for use as an “autologous platelet gel” in wound healing treatment due to its ability to secrete multiple growth factors and the conversion of plasma-rich fibrinogen into a polymer network [9]. Platelet gel preparations have been successful in different wound models (e.g., total skin excision model in rats [10], chronic wound model in diabetic animals, tissue regeneration in patients with clinical oral mucositis and wound healing in patients undergoing surgery for radiological osteonecrosis of the neck and jaws [9,11], etc.). Local delivery of exogenous growth factors plays a significant role in tissue repair [12], while the combination of exogenous growth factors in the treatment process may lead to excessive tissue proliferation, resulting in scarring or fibroproliferative diseases, and their high cost also limits their clinical application [13].

Pearl is a hard object produced in the shell mollusk and the formation process is trigger by an external stimulus. It contains calcium carbonate, protein [14], active peptides [15], amino acids, and many trace elements [16,17]. As a traditional medicine, pre-clinical and clinical studies have shown that pearl powder promotes the maturation of granulation tissue, accelerates the healing process of wounds, causing fewer scars after wound healing. It can be used in the treatment of wound healing-related diseases to treat several wounds, including peptic ulcers, diabetic foot ulcers, oral ulcers, acne, bedsores, burns and scalds. Pearl powder has excellent cytocompatibility, and there is no significant inflammatory reaction after the implantation of pearl fragments into animal tissues. According to reports, the water-soluble components of pearl powder has antioxidant activity [17,18,19,20], which exhibits a vital role in wound healing. Reactive oxygen species (ROS) act as cellular messengers that stimulate the critical process of wound healing. However, when ROS activity increases abnormally, it will cause oxidative damage to cells and slow down the wound healing process. It was demonstrated that pearl powder components provide protective effects against the damage of human keratinocytes (HaCaT) induced by H_2_O_2_ and UV-B [18]. Furthermore, the osteogenic properties and ability to promote collagen expression of the pearl powder has been confirmed [18,21,22]. Bone marrow mesenchymal stem cells (BM-MSC) have been documented as improving wound healing by increasing angiogenesis and promoting cell proliferation [23]. Interestingly, pearl components controlled the signals related to the growth and repair of these cells [22]. It also functioned to stimulate fibroblast mitosis, enhance cell adhesion, collagen deposition and inhibit the tissue inhibitor of the metalloproteinase (TIMP)-1 activity [24]. All these results suggest that pearl powder may be an effective and safe treatment for wounds.

The traditional extraction method of the pearl’s active ingredients is mostly achieved using water, acid or the enzyme extraction process, which has drawbacks such as a low extraction efficiency or the inactivation of the original activity of protein. A supercritical fluid extraction system (SFE) utilizes special properties of a supercritical fluid to achieve the extraction function, which means a state between liquid and gas when the pressure and temperature exceed a specific value. This fluid has a gas-like diffusion coefficient and viscosity, but liquid-like characteristics of density and pressure-dependent solvent capacity [25]. Supercritical fluids have been used as solvents for many applications, such as essential oil extraction [26], metal cation extraction [27], polymer synthesis and particle nucleation [28], particularly in natural product extraction [29]. At present, there are no reports in the literature about using SFE to extract the active ingredients of pearl powder. We hypothesize that it can be accomplished by using an extremely low surface tension of carbon dioxide (CO_2_) in a supercritical state and the great diffusion coefficient, allowing the CO_2_ fluid to possess strong permeability to achieve the extraction of water-soluble components of pearl powder. In addition, the solubility of the fluid can be altered by the addition of a co-solvent. When the desired extract is a polar compound, the incorporation of water or other polar co-solvent (e.g., ethanol) can improve the extraction rate [29]. This may be due to the addition of the co-solvent increasing the density of the mixture and enhancing the physical intermolecular interactions, or it may be due to the creation of specific interactions between the solute and co-solvent molecules, resulting in an overall increase in solubility [30]. Extraction at room temperature, meanwhile, ensures the activity of the protein components is preserved. Therefore, this work explores the feasibility of using supercritical technology to extract the water-soluble components of pearl powder and explores its role in wound healing.

## 2. Materials and Methods

### 2.1. Material

Pearl powder was obtained from Fenix Group Co., Ltd. (Zhuji, China). Dulbecco’s Modified Eagle’s Medium (DMEM) was purchased from Hyclone (Logan, UT, USA). Thiazolyl blue tetrazolium bromide (MTT) was obtained from Sigma-Aldrich (Saint Louis, MO, USA). Fetal bovine serum (FBS) was bought from Gibco (Eugene, OR, USA). Penicillin-streptomycin solution, trypsin/0.25% EDTA were purchased from Shanghai Yeasen Technology Co. Ltd. (Shanghai, China). Bovine serum albumin (BSA) was procured from Yeasen (Shanghai, China). Polyvinylidene difluoride membranes were procured from Millipore (Billerica, MA, USA). Dimethyl sulfoxide (99.9%) were obtained from Sigma-Aldrich (Saint Louis, MO, USA). Bicinchoninic Acid (BCA) Protein Assay Kit was purchased by Solarbio (Beijing, China). Wistar mice were purchased from Slac laboratory animal Co. Ltd. (Shanghai, China). Recombinant human epidermal growth factor (hEGF) (C209) was obtained from Novoprotein (Shanghai, China). A 2-well silicone insert was purchased by ibidi (Martinsried, Germany). Chloral hydrate solid was obtained from sinopharm (Beijing, China). Pluronic F127 (PEO_100_PPO_70_PEO_100_) was purchased from Sigma-Aldrich (Saint Louis, MO, USA). Masson trichrome staining kit was obtained from Solarbio (Beijing, China). Carbon dioxide (99.999% purity) was obtained from Yidong Gas Co., Ltd. (Xiamen, China).

### 2.2. Preparation of Pearl Extract Using SFE

A supercritical fluid extraction system (SFE, Waters Corporation, Shanghai, China) was used for extraction. The whole extraction process was operated only in the autoclave, as the extreme permeability of supercritical fluids for extraction was utilized, rather than the principle involving their strong solubility for non-polar substances. 10 g of pearl crude powder was dissolved in 50 mL Phosphate buffered saline (PBS), and then added in the autoclave with the volume of 0.5 L. The extraction parameters were set as follows. Considering the CO_2_ exhibits supercritical fluid above its critical temperature, 31.0 °C [31], and the inactivation of proteins due to high temperature, we chose the extract temperature as 37 °C. We set the extraction pressure at 25 MPa (above the critical pressure, 7.3773 MPa) [31] and extraction time at 2 h with disposable pumping of CO_2_. When the extraction was finished, the liquid was removed from the autoclave, centrifuged at 5000 rpm for 10 min in a centrifuge and filtered through a 0.22 μm membrane. The collected liquid conponment was water-soluble and it was named SFEPE (Supercritical fluid extraction’s pearl powder extract). The SFEPE solutions were vacuum freeze-dried.

### 2.3. Preparation of Conventional Pearl Powder Water-Soluble Components

The extraction with water was conducted in order to compare the results with the SFEPE. Conventional pearl powder water-soluble components were extracted by soaking in water with constant stirring for 2 h. The collection of the liquid component was the same as the operation in Section 2.2.

### 2.4. Demonstration of Extraction Efficiency

The particle size of the remaining powder after extraction and the protein content in extracts were measured for demonstration of extraction efficiency. Malvern laser particle size analyzer was utilized for the particle size of the remaining powder after extraction. The particle size can reflect the permeation strength of CO_2_ supercritical fluid. It was a reflection of the extraction efficiency, which means the stronger the penetration of supercritical fluid is, the smaller the particle size of residual powder will be. Then, the protein contents in the pearl powder extract were detected by a BCA Protein Assay kit, according to the manufacturer’s instructions. The extracted protein content was taken as an indicator to calculate the extraction rate: the extraction rates = the mass of extraction/original mass of pearl powder.

### 2.5. Cell Culture

The mouse fibroblast cell line L929 and mouse embryonic fibroblasts cell line MEF were cultured in Dulbecco’s Modified Eagle’s Medium (DMEM) with 10% fetal bovine serum (FBS), 100 IU/mL penicillin and 100 g/mL streptomycin, at 37 °C in a humidified atmosphere containing 5% CO_2_.

### 2.6. Cell Viability Test

A cell viability test was used to investigate the effect of extracts on fibroblast growth. L929 or MEF cells were cultured to the appropriate state, digested with trypsin, adjusted to the appropriate number of cells, incubated in the incubator for an appropriate period of time, and 6 different concentrations were set: 50 μg/mL, 25 μg/mL, 10 μg/mL, 5 μg/mL, 1 μg/mL, 0.5 μg/mL, 0.1 μg/mL and 0 μg/mL. Each group was incubated with serum-free medium containing different concentrations of pearl powder extract under normal conditions for 24 h. The culture solution was discarded, 10 μL of 5 mg/mL 3-(4,5-Dimethylthiazol-2-yl)-2,5-diphenyltetrazolium bromide (MTT) solution was added and, after 4 h of incubation in the incubator, the liquid was carefully discarded and 100 μL of DMSO solution was added to each well until it was fully dissolved. The absorbance of each well at 490 nm was measured.

### 2.7. Wound Scratch Assay In Vitro

The wound scratch assay was occupied for in vitro simulation of cell migration during wound healing. L929 and MEF cells were cultured using a cell scratch insert, and after the cells were spread out, the insert was carefully removed with forceps to obtain “scratches” of uniform width in the cell monolayer, and the images of the scratches were taken by inverted fluorescence microscopy. 1 mL DMEM containing 1% FBS with 2 ng/mL GF (growth factor), as a positive control, SFEPE (5, 25 and 50 μg/mL protein concentration) and blank control were added to 6 parallel bores and incubated for 24 h at 37 °C with 5% CO_2_. The images were taken at 24 h, and the cell migration area was used to calculate the migration rate by Image J.

### 2.8. Animal Experiment

The animal model was adopted to further explore the in vivo wound healing activity. Wistar mice with weight of 180–200 g were fed under the protocol of the Animal Care Guidelines of Xiamen University. Given that no significant difference in the activity of the two extracts was found by in vitro activity verification, the SFEPE was chosen to conduct the wound healing experment in vivo. Wistar mice were divided into 4 groups: PBS containing 3% Pluronic F127 as control group; the SFEPE with concentration of 5 mg/mL; 10 mg/mL; and 15 mg/mL. Mice were anesthetized with 10% chloral hydrate solution at a dose of 0.6 mL/100 g body weight injected intraperitoneally. The hair on the back was removed, and then the skin was wiped with iodophor solution and a circular piece of full-thickness skin with a diameter of 1 cm was removed from the shaved area of the back of each mice to make an excision wound. Pearl powder extracts were dissolved in the solution containing 3% Pluronic F127, and was locally delivered at the wound site every day. Vernier calipers were used to measure the wound area at different times after injury.

### 2.9. Histopathological Analysis

Masson’s trichrome staining was utilized for observation of the degree of wound healing. The newly formed skin layers on day 3 after completing various treatments were cut off and fixed in 4% buffered paraformaldehyde for histological staining. 6-μm tissue sections were cut with a microtome, followed by Masson’s trichrome staining. The histological samples were documented on Leica image analyzing system (Leica, Wetzlar, Germany). Morphological features regarding collagen fibers, muscle fibers, granulation tissue and inflammation were analyzed.

### 2.10. Statistical Analysis

Cell viability, cell migration and wound healing rate were performed by expressing the mean ± standard deviation. All available data and figures in this paper were obtained by analyzing and plotting with Origin 8 and GraphPad. Significance was analyzed by using Independent Samples *t*-test, two-tailed Student’s *t*-test and one-way ANOVA. Statistically significant differences were determined as *p* < 0.05.

## 3. Results

### 3.1. Characterization of the Different Extraction in Particle Size and Protein Contents

**Figure 1:**
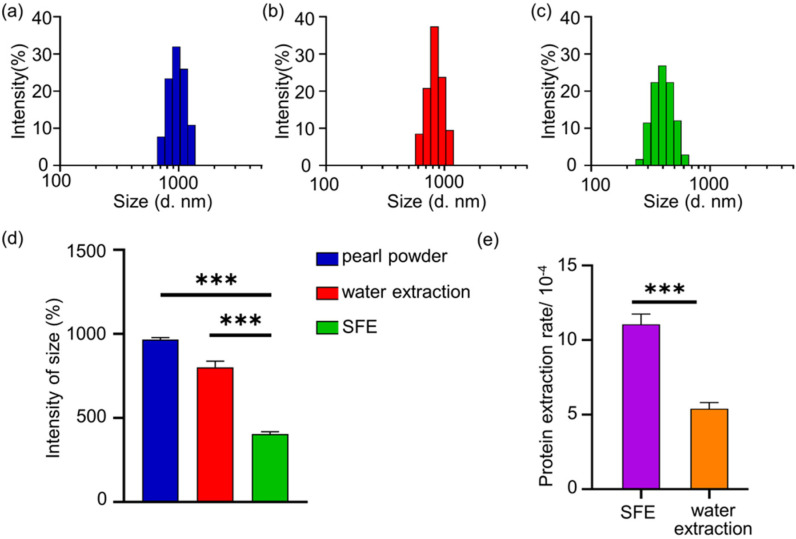
The sizes of pearl powder extracts: (**a**) pearl crude powder, (**b**) conventional water extraction of pearl powder, (**c**) supercritical fluid extraction of pearl powder, (**d**) comparison of particle size of three kinds of pearl powders, (**e**) comparison of the protein extraction rates of the two extraction methods; the protein extraction rates = the mass of extraction/original mass of pearl powder. (*** *p* < 0.001.). Independent Samples *t*-tests were used for analysis.

After feeding into the reaction extraction device for two hours, the particle size of the pearl powder residue extracted by the two different extraction methods was measured. In Figure 1a–d, the original pearl powder particle size measures about 950 nm, the powder particle size obtained after the conventional water extraction method measures 800 nm, while the particle size of the pearl powder after the SFE significantly decreases to approximately 400 nm compared to that of original pearl powder (*p* < 0.001) and conventional water extraction method (*p* < 0.001). It demonstrated that the CO_2_ in the supercritical state in the SFE achieved high penetration strength, exhibiting a strong extraction efficiency. Then the protein concentration was measured by BCA to obtain the protein content in the extracts as 11.06 μg/10 g pearl powder (SFEPE) and 5.40 μg/10 g pearl powder (conventional water extraction), respectively. Meanwhile, we determined the masses of freeze-dried samples of water-soluble components, which were 31.45 μg/10 g pearl powder (SFEPE) and 13.22 μg/10 g pearl powder (conventional water extraction). As observed in Figure 1e, the protein extraction rate in the extract of supercritical fluid extraction system’s pearl powder extraction (SFEPE) was much higher than that in the conventional water extraction method (*p* < 0.001). This result further confirmed that SFE can enhance the extraction efficiency. Since pearls contain a variety of active proteins, subsequent experiments were conducted based on the prerequisite that protein content was used to quantify SFEPE.

### 3.2. Fibroblasts Viability

**Figure 2:**
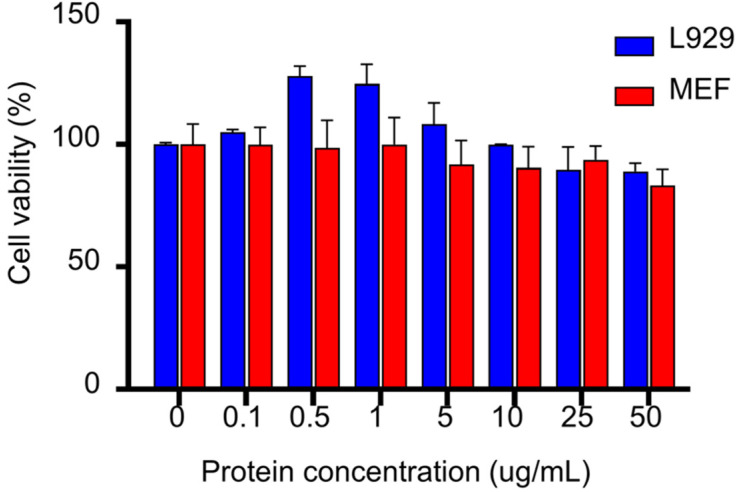
Percentage of survivability about fibroblasts L929 and MEF after 24 h incubation with different protein concentrations of SFEPE.

In order to assess the effect of SFEPE on fibroblast activity, the L929 and MEF cells were exposed to different doses of SFEPE or the conventional water extraction of pearl powder for MTT assay. As shown in Figure 2 and Figure S1, it demonstrated almost no or low cell toxicity on fibroblasts, whether SFEPE or conventional water extraction was used. Hence, the levels of 0–50 μg/mL were selectively used for further wound scratch testing.

### 3.3. Wound Scratch Testing

**Figure 3:**
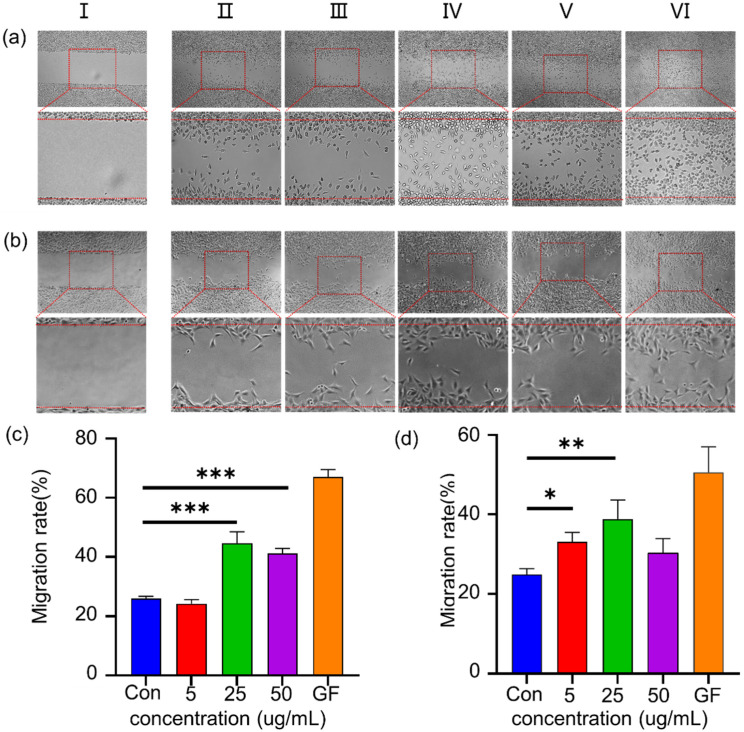
Cell migration to evaluate wound healing in vitro in the scratch assay at 24 h: Images captured at 5× magnification of L929 (**a**) and MEF (**b**) fibroblasts were observed by software in response to the different protein contents of SFEPE. I: scratch at 0 h; II: control group; III: protein concentration 5 μg/mL; IV: protein concentration 25 μg/mL; V: protein concentration 50 μg/mL; VI: GF (growth factor) 2 ng/mL. The statistical data of the wound closure percentage of L929 (**c**) and MEF (**d**) fibroblasts in wound scratch assay was measured. (*** *p* < 0.001, ** *p* < 0.01, * *p* < 0.05).

During the proliferative phase of wound healing, fibroblasts were in the process of migrating, differentiating and synthesizing proteins such as collagen fibers to form granulation tissue. To assess the migration ability of L929 and MEF in wound healing, a wound scratch test in the cell monolayer was conducted to simulate the wound environment. In the SFEPE environment, fibroblasts exhibit enhanced migration capacity. In Figure 3a–c, the migration of L929 and MEF were tested in vitro. The concentration that achieved the strongest migration capacity was 25 μg/mL, whether L929 or MEF. For L929, the protein content of SFEPE in 25 μg/mL and 50 μg/mL showed higher lever proliferation and migration, in comparison with the control group (*p* < 0.001). While in the MEF group, 5 μg/mL (*p* < 0.05) and 25 μg/mL (*p* < 0.01) protein content exhibited the ability to promote migration, respectively. In the quantitative analysis of the migration rate of fibroblasts, it was also found that the migration rate of cells in the group containing SFEPE was stronger than that of the control group. These results implied that SFEPE was capable of promoting fibroblasts migration, which might exhibit the capacity for accelerated wound healing. Significantly, SFEPE and conventional water extraction exhibited a consistent ability to promote cell migration (Figure S2).

### 3.4. Wound Healing Activity In Vivo

**Figure 4:**
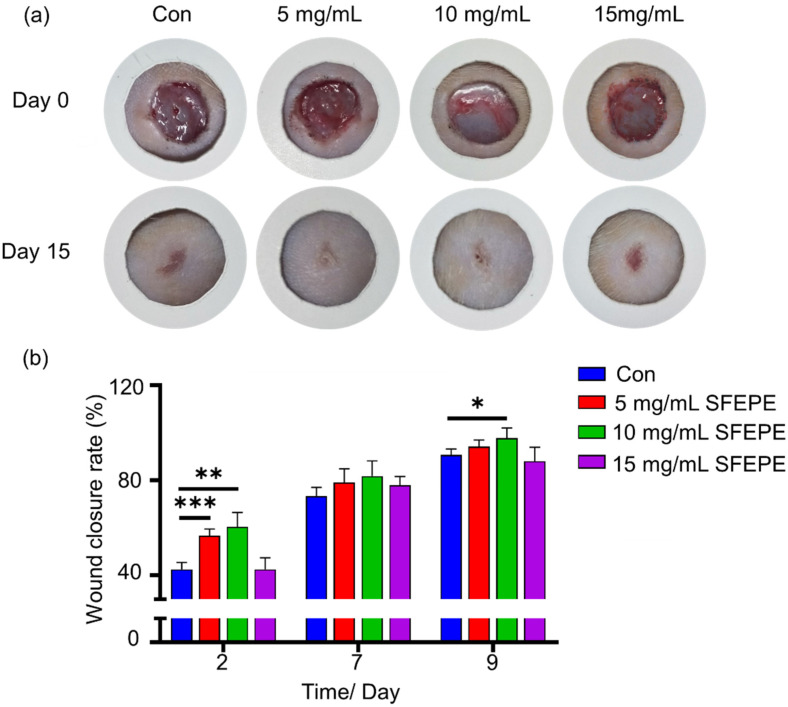
In vivo wound healing in Wistar mice, with a full thickness skin excision wound model. (**a**) Image acquisition of wounds at the time of wound formation and two weeks later. (**b**) Wound area was collected on days 2, 7, and 9 after wound formation to quantify the wound healing rate. (*** *p* < 0.001, ** *p* < 0.01, * *p* < 0.05).

The results of wound healing rates in the injury healing experiment with full skin excision in rats are shown in Figure 4. The three treatment groups were the control group; 5 mg/mL; and 10 mg/mL and 15 mg/mL. As Figure 4a,b shows, the SFEPE with a concentration of 5 mg/mL and 10 mg/mL are shown to promote wound healing ability. Respectively, the 10 mg/mL SFEPE group showed significant wound healing effect, exhibiting 99.91 ± 0.16% healing rate. The difference of wound closure rate between the group with and without 5 mg/mL was ~14.18% at day 2, ~5.78% at day 7 and ~3.54% at day 9. SFEPE with 10 mg/mL showed a higher wound closure ratio compared with the control group with a difference of about 17.86%, 8.48% and 7.15% at day 2, 7, and 9 respectively. Interestingly, the dosage of 15 mg/mL exhibited weaker wound healing effects. We hypothesized that the dosage represented cytotoxicity to derma. These observations demonstrated that SFEPE exhibited facilitating wound healing ability.

### 3.5. Histological Analyses

**Figure 5:**
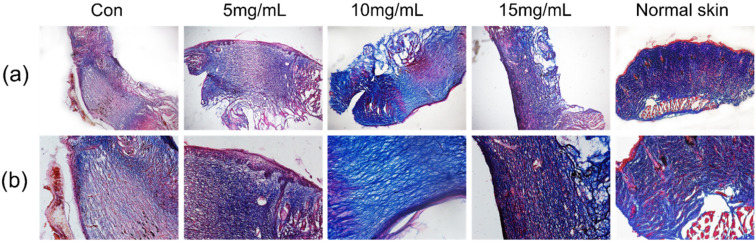
Image-captured tissue slices with Masson’s trichrome staining at 9 d post-wounding, under magnification of 5× (**a**) and 10× (**b**), respectively.

Each treatment group demonstrated visible granulation tissue during skin tissue repair and, in normal tissues, collagen fibers were regularly distributed, the epidermis was intact, and there were an abundance of hair follicles as shown in Figure 5. SFEPE-treated groups achieved better efficiency of re-epithelialization, exhibited more abundant collagen fibers, and more intact epidermises.

## 4. Conclusions

As a traditional mineral medicine, pearls have been proven to be effective in calming the mind, brightening the eyes, removing blemishes and beautifying the face, over thousands of years of clinical use in China. However, the mechanism of pearl powder on wound repair in the body has not yet been scientifically proven. Meanwhile, there is no detailed report on the relationship between the extraction method of pearl powder and the activity of the extracts, so an efficient and safe extraction method and the acquisition of highly active extracts are the keys to achieve efficient utilization of pearl powder.

Supercritical fluid extraction techniques offer unique advantages in the extraction of bioactive compounds and overcome many of the limitations present in other extraction methods. Their powerful permeation properties and optimization through parameters can provide faster extraction rates of active ingredients from pearl powder. Given their powerful permeation properties, we envisage that SFE has a high potential to be used for the extraction of active substances from pearl powder. We also included a PBS buffer in extract system, which we thought would change the solubility of active ingredients in the extraction kettle to improve the extraction rate. More importantly, compared to ethanol, it does not destroy the structure of the active protein, which was the reason that we didn’t choose the ethanol as a cosolvent; although it is more often used as a cosolvent for the extraction of polar components, and there are almost no reports of using PBS as a co-solvent.

We conducted the extraction on the basis of protein being an important active ingredient in pearl powder and it was demonstrated that the protein content obtained by SFE was significantly higher than that of conventional aqueous extracts for the same extraction time, while the particle size distribution of the residual powder extracted by SFE was more uniform and significantly reduced.

After normal wound generation, fibroblasts proliferate profusely, followed by the synthesis and secretion of large amounts of collagen fibers and matrix components by fibroblasts, and together with new capillaries they form granulation tissue. In the late stage of repair, they participate in the alteration of the repaired tissue by secreting collagenase. Fibroblasts have a vital role in the process of wound tissue repair, therefore, an attempt was made to investigate the position of SFEPE in wound healing by studying the role of SFEPE on fibroblasts. In the fibroblasts viability assay, it was demonstrated that SFEPE has the ability to increase L929 activity and promote L929 proliferation at specific concentrations. Cell migration assays demonstrated no or low cell toxicity on fibroblasts. Significantly, SFEPE was revealed to have a more significant potential to promote fibroblast migration in low concentration; it agreed with a number of previous results [32,33]. However, its promoting mechanism of cell proliferation remains to be studied.

The strongest pro-migration effect was observed at 25 μg/mL of SFEPE for both L929 and MEF cells. Growth factors are endogenous-signaling molecules mediating cellular responses during tissue repair [34], which regulate a variety of cellular behaviors, such as proliferation, migration and differentiation, showing great potential in wound healing therapy [35]. Although the experimental data demonstrating that pearl powder extract had a weaker ability to contribute to fibroblast migration than growth factors, the utilization of growth factors might trigger tissue over-proliferation and excessive healing concerns. For example, a transforming growth factor (TGF)-β produces a pronounced tissue response, manifested by an increase in new connective tissue and collagen content [36]. However, the rapid and efficient repair response explains this abnormal regulation, with which excessive deposition of extracellular matrix might cause fibro-proliferative disorders in human skin, resulting in scarring at the site of trauma [12,37]. Meanwhile, the application of growth factors was reported to increase the risk of cancer [13,38]. Furthermore, their short effective half-life and low bio-stability limited their clinical application [35]. Based on these characteristics, in comparison with growth factor therapy, the pearl powder extract in this study might provide a milder approach in wound healing treatment.

The activity investigation revealed no significant difference in the activity of the two extracts with the same protein content. The good biocompatibility of the extracts obtained using SFE was demonstrated. It is worth mentioning that using aqueous extraction, the extraction efficiency was much lower than the method of SFE. When using acid or enzymatic hydrolysis methods, non-essential impurities are introduced in the extracts, which affect their activity to some extent. In addition, the complex process of impurities removal also increases the extraction cost, while the solvent is residue-free, environmentally friendly and reduces the process cost with the supercritical extraction technique.

The therapeutic effect of pearl powder extract was studied in an animal model of total skin excision wounds. It can be seen from the wounds (Figure 4a) that the granulation tissue repair was faster in the pearl powder group compared with the control group. Masson staining was performed, and as seen in Figure 5, the blank group had a weak collagen fiber formation capacity and staining displayed sparse cerebral tissues. In contrast, some of the pearl groups were observed to have accessory organogenesis and the collagen fibers were regularly distributed with higher content.

## 5. Discussion

The mechanism of wound healing by pearl powder is currently being studied. Chen et al. explored the efficacy of different particle sizes of pearl powder [32]. Nanosizing was found to be a way to improve the solubility of active ingredients, enhance therapeutic effect and improve safety. Furthermore, Li et al. investigated the mechanism of wound healing via water-soluble components of pearl powder and demonstrated its activity in promoting cell migration, as well as promoting type III collagen expression [24]. Based on previous studies, we designed a new method for extracting the active ingredients of pearl powder with supercritical fluids and verified the activity of the extracts from the new method in wound healing. Our results were almost identical to theirs, except that the effect of promoting cell proliferation was not significant. We speculate that it may be caused by some new components extracted by new methods.

However, there are still some questions. The literature reports that pearl powder contains calcium carbonate, protein, amino acids and trace elements, etc. [16], so what are the ingredients in the extract and what is the proportion of each ingredient? In this paper, a new method was used to extract pearl powder components. What are the component differences between supercritical extracts and those extracted by other methods? Which specific components are responsible for the wound healing activity? What is the exact mechanism of the relevant components in the active pathway? Another important issue is that, in this study, the wound healing activity of supercritical fluid pearl powder extract was initially verified, but the process parameters of the extraction process were not optimized. The differences in the composition and activity of the extracts under different parameters were also not investigated in depth at this point. These issues remain to be resolved in future work.

In conclusion, this project achieved the extraction of the active ingredients of pearl powder by a new process, specifically, the extraction of the active ingredients of pearl powder using supercritical fluid with very strong permeability to achieve high efficiency. The extract from this process was further explored and we confirmed its activity in wound healing.

## Data Availability

Not applicable.

## Supplementary Materials

The following are available online at https://www.mdpi.com/article/10.3390/ma14164458/s1, Figure S1: Percentage of survivability about fibroblasts L929 after 24 h incubation with different protein concentration of SFEPE and conventional water extract, Figure S2: Cell migration to evaluate wound healing in vitro in the scratch assay at 24 h: Images captured at 5× magnification of L929 was observed software in response to different protein content of extract.
