# Supplementary material for: Efficacy of Water-Soluble Pearl Powder Components Extracted by a CO2 Supercritical Extraction System in Promoting Wound Healing
Source: Materials (Basel). 2021 Aug 9;14(16):4458. doi: 10.3390/ma14164458 (PMC8399097; doi:10.3390/ma14164458)
Supplement: Supplementary file 1 [file materials-14-04458-s001.zip › materials-1264371-supplementary.pdf]

Supplementary Materials

Efficacy of Water-Soluble Extracts of Pearl Powder by CO2 supercritical extraction system in Promoting Wound Healing

Minting Liu[a,#], Junjun Tao[b,#], Hongchen Guo[b], Liang Tang[b], Guorui Zhang[b], Changming Tang[b], Hu Zhou[a], Yun-Long Wu[a], Huajun Ruan[b,*] and Xian Jun Loh[c,*]

Cell viability test： Cell viability test was investigated for the effect of extracts on cell activity. L929 cells were cultured to the appropriate state, digested with trypsin, adjusted to the appropriate number of cells, incubated in the incubator for an appropriate period of time, and 6 different concentrations of SFEPE and conventional water extract were set: 0 μg/mL, 0.45 μg/mL, 0.9 μg/mL, 1.8 μg/mL, 3.5 μg/mL, 7.5 μg/mL, 15 μg/mL,30 μg/mL, 60 μg/mL, 120 μg/mL. Each group was incubated with se-rum-free medium containing different concentrations of pearl powder extract under normal conditions for 24 h. The culture solution was discarded, 10 μL of 5 mg/mL 3-(4,5-Dimethylthiazol-2-yl)-2,5-diphenyltetrazolium bromide (MTT) solution was added, and after 4 h of incubation in the incubator, the liquid was carefully discarded and 100 μL of DMSO solution was added to each well until it was fully dissolved. The absorbance of each well at 490 nm was measured.

Wound scratch testing: The wound scratch assay was occupied for *in vitro* simulation of cell migration during wound healing. L929 cells were cultured using a cell scratch insert, and after the cells were spread out, the insert was carefully removed with forceps to obtain "scratches" of uniform width in the cell monolayer, and the images of the scratches were taken by inverted fluorescence microscopy. 0.5 mL DMEM containing 1% FBS with 2 ng/mL GF (growth factor), as a positive control, SFEPE and conventional water extraction (5, 25 and 50 μg/mL protein concentration) and blank control was added to 6 parallel bores and incubated for 24 h at 37 °C with 5% CO2. The images were taken at 24 h, and the cell migration area was used to calculate the migration rate by Image J.

Statistical Analysis: Significance was analyzed by using Independent Samples t-Test, Two-tailed Student's t-Test and one-way ANOVA. Statistically significant differences were determined as $p < 0.05$.

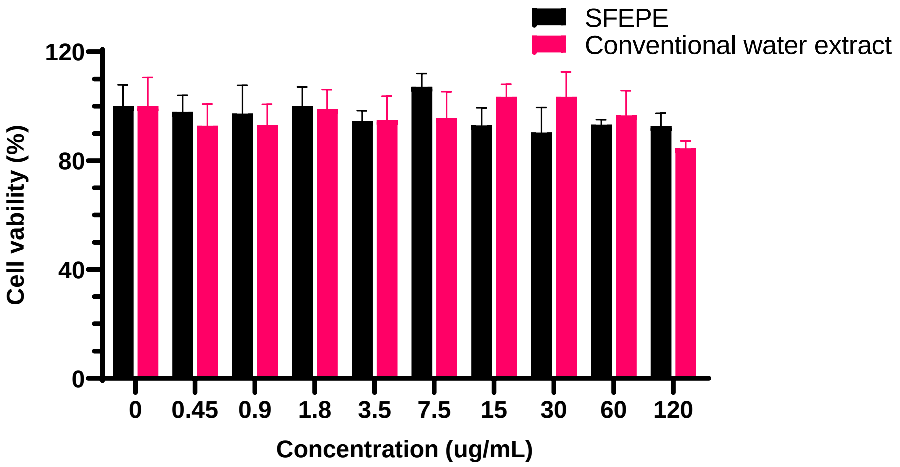

Figure S1. Percentage of survivability about fibroblasts L929 after 24h incubation with different protein concentration of SFEPE and conventional water extract.

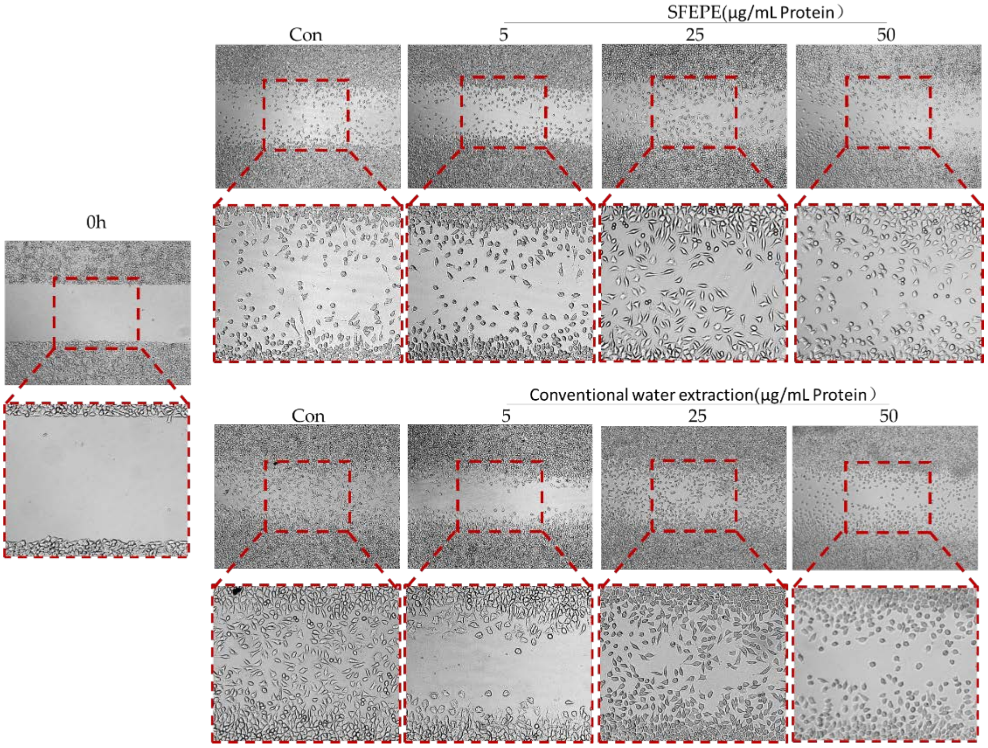

Figure S2. Cell migration to evaluate wound healing in vitro in the scratch assay at 24 h: Images captured at 5× magnification of L929 was observed software in response to different protein content of extract.
